# Silencing of the Mitogen-Activated Protein Kinases (MAPK) *Fus3* and *Slt2* in *Pseudocercospora fijiensis* Reduces Growth and Virulence on Host Plants

**DOI:** 10.3389/fpls.2018.00291

**Published:** 2018-03-13

**Authors:** Francis Onyilo, Geoffrey Tusiime, Jaindra N. Tripathi, Li-Hung Chen, Bryce Falk, Ioannis Stergiopoulos, Wilberforce Tushemereirwe, Jerome Kubiriba, Leena Tripathi

**Affiliations:** ^1^International Institute of Tropical Agriculture, Nairobi, Kenya; ^2^Department of Agricultural Production, Makerere University, Kampala, Uganda; ^3^National Agricultural Research Laboratories, Kampala, Uganda; ^4^Department of Plant Pathology, University of California, Davis, Davis, CA, United States

**Keywords:** *Pseudocercospora fijiensis*, mitogen-activated protein kinase, *Fus3*, *Slt2*, pathogenicity

## Abstract

*Pseudocercospora fijiensis*, causal agent of the black Sigatoka disease (BSD) of *Musa* spp., has spread globally since its discovery in Fiji 1963 *to* all the banana and plantain growing areas across the globe. It is becoming the most damaging and economically important disease of this crop. The identification and characterization of genes that regulate infection processes and pathogenicity in *P. fijiensis* will provide important knowledge for the development of disease-resistant cultivars. In many fungal plant pathogens, the *Fus3* and *Slt2* are reported to be essential for pathogenicity. *Fus3* regulates filamentous-invasion pathways including the formation of infection structures, sporulation, virulence, and invasive and filamentous growth, whereas *Slt2* is involved in the cell-wall integrity pathway, virulence, invasive growth, and colonization in host tissues. Here, we used RNAi-mediated gene silencing to investigate the role of the *Slt2* and *Fus3* homologs in *P. fijiensis* in pathogen invasiveness, growth and pathogenicity. The *PfSlt2* and *PfFus3* silenced *P. fijiensis* transformants showed significantly lower gene expression and reduced virulence, invasive growth, and lower biomass in infected leaf tissues of East African Highland Banana (EAHB). This study suggests that *Slt2* and *Fus3* MAPK signaling pathways play important roles in plant infection and pathogenic growth of fungal pathogens. The silencing of these vital fungal genes through host-induced gene silencing (HIG) could be an alternative strategy for developing transgenic banana and plantain resistant to BSD.

## Introduction

*Pseudocercospora fijiensis*, causal agent of black Sigatoka disease (BSD) in *Musa* spp. (banana and plantain), was first recognized in 1963 in the South-Eastern Coast of Viti Levu in Fiji (Mourichon et al., 1997; Marin et al., 2003; Churchill, 2011). Almost 30 years later, BSD was reported in Honduras from where it spread to Guatemala, Southern Mexico, Panama, Ecuador, and Peru. In Southeast Asia, it is found in the Philippines, Taiwan, and Indonesia. In Africa, BSD was first reported about 25–30 years ago in Gabon and Zambia (Churchill, 2011). Since then, BSD has spread to sub-Saharan countries in the West African coast and to the Eastern African countries (Mourichon et al., 1997; Ploetz, 2001; Marin et al., 2003; Churchill, 2011).

*Pseudocercospora fijiensis* is one of the most damaging and economically important pathogens of *Musa* spp. worldwide (Farr et al., 1995; Stewart et al., 1999; Carlier et al., 2000). Attempts to control BSD include frequent application of fungicides and cultural practices such as the removal of infected leaves and proper spacing and drainage in plantations (Ploetz, 2001). Fungicide control of BSD in Central America is responsible for approximately 27% of the retail price of bananas (Stover, 1980, 1986; Stover and Simmons, 1987). The cost of chemical control of BSD is estimated about US$400 to US$1,400 per hectare (Stover, 1986; Pasberg-Gauhl et al., 2000; Ploetz, 2000; Arias et al., 2003; Churchill, 2011), and smallholder farmers cannot afford fungicides and are therefore more prone to losses due to BSD. Also, *P. fijiensis* develops resistance to fungicides after many sprays and therefore, better strategies are needed to efficiently control this disease (Stover, 1990).

*Pseudocercospora fijiensis* reproduces sexually by means of ascospores that are mainly produced during the later stages of disease and asexually through conidia produced during the early stages. Ascospores are the main way of long-distance dispersal of *P. fijiensis* between plantations and into new areas (Ploetz, 2001; Marin et al., 2003; Agrios, 2005). The spores germinate within 2–3 h under high humidity and temperatures greater than 20°C, and then enter the host through the stomata openings within 48–72 h (Stover, 1980). The fungal hyphae then grow inside the leaf, colonizing the intercellular spaces and killing plant cells. After infection, the pathogen emerges from the stomata and will develop conidiophores that can start the new cycle of infections (Churchill, 2011). Streaks that usually appear first near the leaf apex and along the leaf margin are a sign of infection. Initial symptoms are seen only at 10–30 days after infection. Diseased leaves will become sources of inoculum for new infections (Meredith, 1970; Carlier et al., 2000; Marin et al., 2003). Aggressiveness of *P. fijiensis* is directly related to environmental conditions; BSD is more pronounced when relative humidity is greater than 80% and when temperature is above 23°C (Fouré, 1994; Gauhl, 1994; Torrado-Jaime and Castaño-Zapata, 2008).

Though efforts are underway to develop BSD host resistance in banana and plantain through conventional breeding, genetic engineering could be an alternative approach for developing resistant cultivars. One possible means might be to use RNA interference (RNAi) to target fungal genes responsible for regulating plant infection, invasive growth, and pathogenicity of *P. fijiensis*. Transgenic banana resistant to fusarium wilt disease were developed through posttranscriptional silencing of fungal genes *velvet* and *Fusarium transcriptional factor 1* (Ghag et al., 2014).

**FIGURE 1:**
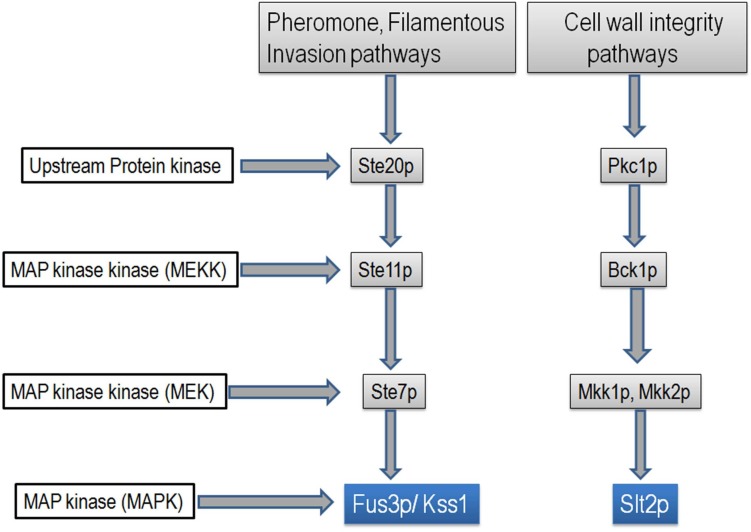
Schematic diagram showing pheromone, filamentous invasion, and cell-wall integrity pathways of the MAP kinase cascade of *Saccharomyces cerevisiae.* Diagram is adopted from (Errede et al., 1995; Gustin et al., 1998).

A family of serine/threonine protein kinases known as mitogen-activated protein kinases (MAPKs) are involved in the transduction of a variety of extracellular signals and the regulation of different developmental processes. The yeast extra cellular signal-regulated kinase (YERK1) is the most thoroughly investigated MAPK. The MAPK cascade in *Saccharomyces cerevisiae* has three protein kinases that act in series; a MAP kinase kinase kinase (MAPKKK or MEKK), a MAP kinase kinase (MAPKK or MEK) and finally MAP kinase (MAPK) (Marshall, 1994; Cobb and Goldsmith, 1995). Upon activation of the cascade, MAPKKK phosphorylates the MAPKK, which in turn phosphorylates MAPK (**Figure 1**). The MAPK cascades in fungi regulate transcription factors by MAPK mediated phosphorylation (Errede et al., 1993, 1995). The MAPKs, *Fus3*, and *Slt2* appear to be involved in pathogenicity of fungi (Mayorga and Gold, 1999; Xu, 2000). *Fus3* regulates pheromone response and invasion pathways, while *Slt2 is* involved in the cell-wall integrity pathway (Mayorga and Gold, 1999; Xu, 2000).

A number of YERK1 proteins have been shown to be involved in the formation of infection structures such as appressoria and the invasive growth of fungal plant pathogens, such as the maize pathogen, *Ustilago maydis* (Mayorga and Gold, 1999; Muller et al., 1999). In addition, MAPKs take part in signal transduction pathways that are activated in regulation of growth and development (Alonso-Monge et al., 2001). The *Fus3/Kss1*-type related gene *MaMk1*, identified in *Metarhizium acridum*, encodes a member of the YERK1 subfamily, which is known for regulating appressorium formation and insect cuticle penetration (Jin et al., 2014). In several fungal plant pathogens including *Zymoseptoria tritici*, *Puccinia striiformis* f. sp. *tritici*, *Fusarium oxysporum*, and *F. proliferatum*, *Fus3/Kss1* homologs are shown to be responsible for colonization in mesophyll tissue, growth, spore formation, penetration, and virulence (Mendgen et al., 1996; Cousin et al., 2007; Guo et al., 2011; Zhao et al., 2012).

Likewise, MAPK *Slt2* has been well studied in *S. cerevisiae* and is known to be required for cell-wall integrity (Xu, 2000). In *Z. tritici* and the entomopathogenic fungus *Beauveria bassiana, Slt2* homologs are well known for their roles in invasive growth and virulence (Mehrabi et al., 2006; Luo et al., 2012) and in *Alternaria alternata*, it is crucial for conidial formation, hyphal elongation and fungal pathogenicity (Yago et al., 2011). Silencing of *PsMpk1*, a *Slt2* type MAPK in the oomycete *Phytophthora sojae*, showed loss in pathogenicity on susceptible soybean host plants, with triggered enhanced cell death (Li et al., 2014). Defects in fungal growth, zoosporogenesis, and increased hypersensitivity to cell-wall degrading enzymes were also reported (Li et al., 2014).

*Pseudocercospora fijiensis* and *Z. tritici* are phylogenetically related, and since *Fus3* and *Slt2* are known to be responsible for regulating the host penetration, invasive growth, and pathogenicity of *Z. tritici*, it is possible that *Fus3* and *Slt2* are important pathogenicity factors for *P. fijiensis* as well. Therefore, to study the roles of *Fus3* and *Slt2* in pathogenicity of *P. fijiensis*, we silenced these genes and tested the transformants on young potted tissue-culture plants of East African Highland Banana (EAHB) cultivar ‘Nakitembe’ for disease development. This study confirmed that the *Slt2* and *Fus3* MAP kinase signaling pathways are important for plant infection, invasive growth and pathogenicity of *P. fijiensis* in EAHB. Therefore, targeting *PfFus3* and *PfSlt2* could contribute to developing resistant varieties of banana and plantain against *P. fijiensis* causal agent of BSD.

## Materials and Methods

### *Pseudocercospora fijiensis* Culture Isolation and Confirmation

The *P. fijiensis* culture used in this study was isolated from infected leaves of the banana cultivar ‘Nakitembe’ as described by Onyilo et al. (2017). Genomic DNA was extracted from mycelia following the protocol described by Mahuku (2004) with some modifications. The *P. fijiensis* isolate was confirmed by PCR using *P. fijiensis*-specific primers (MF137 GGCGCCCCCGGAGGCCGTCTA and R635 GGTCCGTGTTTCAAGACGG) based on ITS region (Johanson and Jeger, 1993). The cultures of *P. fijiensis*, *P. musae*, and *P. eumusae* collected from CBS-KNAW, the Fungal Biodiversity Centre in Netherlands, were used as controls.

### Plasmid Construct Preparation and Molecular Characterisation

#### PCR Amplification of *PfFus3* and *PfSlt2* Genes

The fragments of homologs of *Fus3* and *Slt2* from *P. fijiensis* (i.e., 358 bp of *PfFus3* and 264 bp of *PfSlt2)* were amplified from genomic DNA of *P. fijiensis* using gene-specific primers. FUS35′: CGCACGCACATTACCTACACCCTC, FUS33′: CATGGAATGGTCGAAGGGTGTG and SLT25′: CAATGATTTGGAGAGAGAGC, SLT23′: GCCACTACCCATGCATTTCTTC primers were designed for *P. fijiensis* based on CIRAD86 MAP kinase accession numbers XM_007929802.1 and XM_007927722.1.

The PCR reaction mixture contained 10 μM each of the forward and reverse primers (0.5 μl), AmpliTaq^®^ DNA polymerase (0.25 μl), (Applied Biosystems, United States), 10× buffer with 15 mM MgCl2 (2.5 μl), (Applied Biosystems, United States), 10 μM deoxyribonucleotides (dNTP) (0.5 μl), 1 μl (100 μg) of genomic DNA of *P. fijiensis*, adjusted with water to 25 μl final volume. The conditions used were the following: initial denaturation at 95°C for 5 min, 34 cycles of denaturation at 95°C for 30 s, annealing temperature at 55°C for *Fus3* and 52°C for *Slt2* for 30 s, and extension at 72°C for 1 min, followed by a final extension at 72°C for 5 min and storage at 12°C. Amplicons were separated by electrophoresis on 0.8% (w/v) agarose gels. PCR products of *Fus3* (358 bp) and for *Slt2* (264 bp) were isolated from the gel and purified using Zymoclean^TM^ gel DNA recovery kit following the manufacturer’s protocol.

**FIGURE 2:**
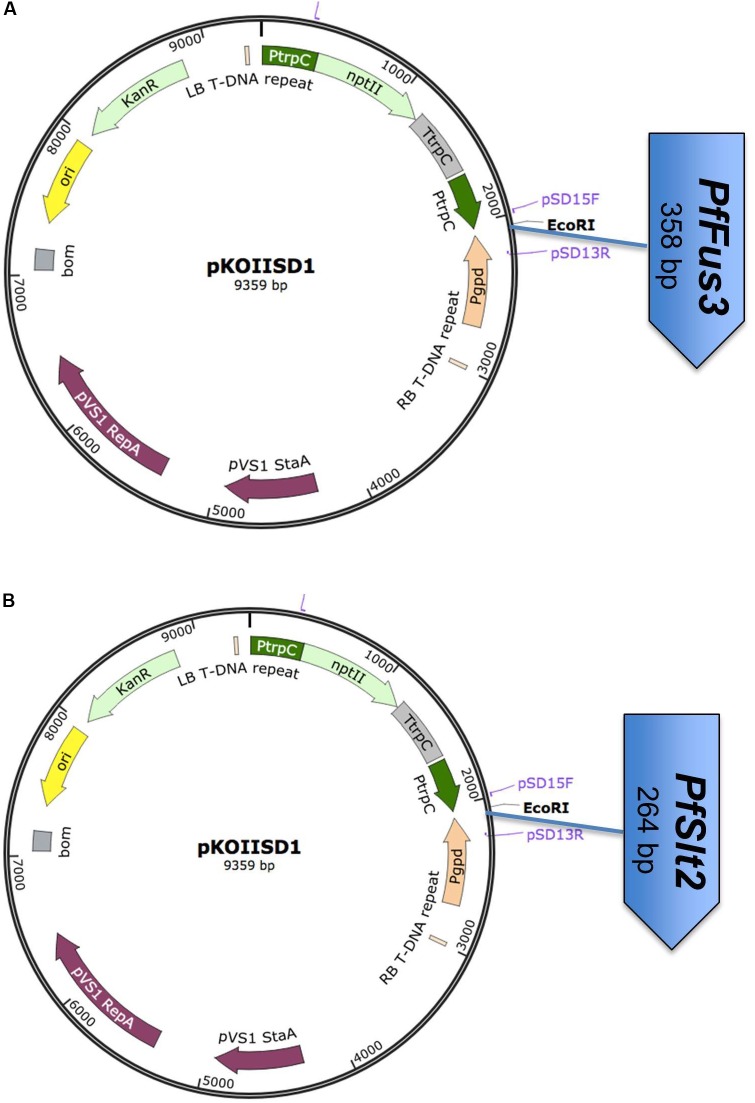
Schematic diagram of RNAi plasmid constructs. **(A)** pKOIISD1-*PfFus3;* **(B)** pKOIISD1-*PfSlt2.* PtrpC, promoter *trpC* of *Aspergillus nidulans*; Pgpd, promoter *gpd* of *Aspergillus nidulans*; *npt*II, neomycin phosphotransferase II gene; LB, Left Border of T-DNA; RB, Right Border of T-DNA fragment.

The purified *Fus3* (358 bp) and *Slt2* (264 bp) fragments were ligated into pKOIISD1 plasmid at the EcoRI site and constructs were named as pKOIISD1-*PfFus3* and pKOIISD1-*PfSlt2* (**Figures 2A,B**). Plasmid constructs pKOIISD1-*PfFus3* and pKOIISD1-*PfSlt2* were confirmed for the presence and orientation of inserts by PCR and sequencing, respectively.

#### Transfer of Plasmid Constructs to *Agrobacterium tumefaciens*

After validation, the plasmids pKOIISD1-*PfFus3* and pKOIISD1-*PfSlt2* were transferred to *Agrobacterium tumefaciens* strain *AGL1* according to Onyilo et al. (2017). Colony PCR was then performed to validate the presence of *PfFus3* and *PfSlt2* in transformed *AGL1*, using primer pairs, pSD15′ (CTTTAAGTTCGCCCTTCCTC) and pSD13′ (GTTGACAAGGTCGTTGCGT) designed from the pKOIISD1 vector. PCR reaction mixtures contained 10 μM primers (0.5 μl each of pSD15′ and pSD13′), AmpliTaq^®^ DNA polymerase (0.25 μl, Applied Biosystems, United States), 10× Buffer with 15 mM MgCl2 (Applied Biosystems, United States), 10 μM deoxyribonucleotides (dNTP), *AGL1*, and water in a total volume of 25 μl. PCR program cycles used were the following: initial denaturation at 95°C for 5 min and then 34 cycles of denaturation at 95°C for 30 s, annealing at 55°C for 30 s and extension at 72°C for 1 min, followed by final extension at 72°C for 5 min and storage at 12°C. The amplified PCR product was separated by electrophoresis on agarose gels.

#### Transformation of *P. fijiensis* and Molecular Characterization

The PCR-positive colonies of transformed *Agrobacterium tumefaciens* strain *AGL1* containing pKOIISD1-*PfFus3* or pKOIISD1-*PfSlt2* were maintained and used to transform *P. fijiensis* through *Agrobacterium tumefaciens*-mediated transformation as described by Onyilo et al. (2017).

Genomic DNA was isolated from plugs of mycelia of transformed *P. fijiensis* grown in V8 juice medium at 25°C following extraction protocol as described above. The transformed *P. fijiensis* was validated for presence of *PfSlt2* and *PfFus3* by PCR using primer pairs pSD15′ and pSD13′. Wild-type (WT) untransformed *P. fijiensis* was used as control.

After validation by PCR, three transformants of *P. fijiensis* with silenced *PfFus3* (i.e., *PfFus3-5, PfFus3*-11, *PfTFus3-12*) and *PfSlt2* (i.e., *PfSlt2-1*, *PfSlt2-11, PfSlt2-12*) were selected randomly for further analysis for gene expression, pathogenicity assays, growth and biomass estimations.

#### Evaluation of *Fus3* and *Slt2* Expression in *P. fijiensis* Transformants

Total RNA was extracted from wild-type and transformed *P. fijiensis* using TRIzol Reagent following the protocol provided by Ambion RNA life technologies. Total RNA was purified by RNA clean and concentrator^TM^ kit according to the Zymo research Corp manual. cDNA was prepared using Maxima first strand cDNA kit (Thermo Fishers Scientific, Inc.).

### Gene Expression Assay

Quantitative reverse transcription polymerase chain reaction (qRT-PCR) was performed to determine expression levels of *PfFus3* and *PfSlt2* by relative Quantitation (i.e., Threshold cycle; *C*t). Three different transformants of *PfFus3* (i.e., *PfFus3-5, PfFus3*-11, *PfFus3-12.*) and *PfSlt2* (i.e., *PfSlt2-1*, *PfSlt2-11, PfSlt2-12*) were randomly selected for qRT-PCR. Three technical replicates for each and WT control were used. The β*-tubulin* gene was used as reference gene and a non-transformed wild-type *P. fijiensis* and non-template as controls. Gene-specific primer pairs FUS35′: TGCGAATTTCACGTCTCTGC and FUS33′: TGTGGTGTGTTTGCGAATGG; SLT25′: TCGATGCCATGCGACAATAG, and SLT23′: CCCTCTTCACGATGCAACAAC for *PfFus3* and *PfSlt2* were used, respectively. Primer pairs β-tubulin5′: ATACACACCGCATCAACGAC and β-tubulin3′: ATGAACGATCTCGCATTC from sequence accession number XM_007921924.1 were used for reference gene β*-tubulin*. The reaction mixture contained: Maxima SYBR Green/ROX qPCR Master mix (2×) Thermo Scientific, 300 nM β- tubulin, *Slt2* and *Fus3* primers, 100 ng/μl DNA in a total reaction volume of 12 μl. qRT-PCR cycles used were as follows: 50°C for 2 min, 95°C for 10 min, 95°C for 15 s, 60°C for 30 s annealing, 72°C for 30 s 40× cycles, followed by melting curve stages. For a given serial dilution of DNA/cDNA, dilution factor of 1/10 was used. Graph Pad Prism software version 5 and Microsoft Excel 2007 were used in generating linear regression curves, for evaluating primer specificity and efficiency.

### Virulence Assays for *PfFus3* and *PfSlt2* Transformants

Mycelial fragments from wild-type *P. fijiensis* and silenced *P. fijiensis* strains (i.e., *PfFus3-5, PfFus3*-11, *PfTFus3-12*, *PfSlt2-1*, *PfSlt2-11*, and *PfSlt2-12)* were cultured in 200 ml of rich medium (yeast extract 10 g, glucose 30 g in 1 L of double-distilled water) containing 100 μg/ml ampicillin and incubated at room temperature for 5 to 10 days at 150 rpm. Equal amounts of mycelia were macerated in a sterile mortar and filtered using double-layered cheesecloth. Mycelial fragments were resuspended in 10% rich medium containing 1% tween 20, counted using a hemocytometer, and adjusted to 10^4^ mycelia fragments/mL. The inoculum was applied on the abaxial side of leaves of 3-month-old potted tissue culture plants of EAHB cultivar ‘Nakitembe’ with the help of a fine paintbrush. The inoculated plants were incubated under high humidity in a humid chamber covered with clear polythene. Misting of plants was done three times every day for a period of 3 days in order to maintain 80 to 90% humidity in the humid chamber. Disease severity was estimated by counting the number of necrotic lesions per 2 cm^2^ areas at four different points on the inoculated leaf. These counts were used to determine the mean necrosis and the disease stage scores were assessed according to Onyilo et al. (2017). Three plant replicates with three leaves per plant were used in each experiment and repeated three times.

To determine the fungal mycelia growth in leaf tissues ca. 3 cm leaf disks were neatly cut with sterile surgical blades at 45 days post inoculation and stained as described by Onyilo et al. (2017). The slides were observed for fungal plant infections, invasive growth, and colonization using a COSLAB light microscope, and pictures were taken using a digital camera MDCE-5C (ISO 9001 Co) and analyzed in Optika Vision Lite 2.1 Software.

### Biomass Quantification of *P. fijiensis* in Leaf Tissue

Infected leaves were harvested at 45 days post inoculation. Genomic DNA was extracted from 1 g of samples of pure *P. fijiensis* culture, plant leaves infected with *P. fijiensis* transformants (i.e., *PfFus3-5, PfFus3-11, PfTFus3-12, PfSlt2-1, PfSlt2-11* and *PfSlt2-12)*, wild-type *P. fijiensis*, and non-infected banana leaf samples according to the protocol described by Mahuku (2004). The DNA samples were treated with 1 μl of 10 mg/μl RNase A in a total volume of 50 μl at 37°C for 45 min. The reaction was terminated at 65°C for 10 min. DNA was re-precipitated by adding 150 μl of 100% absolute ethanol and resuspended in 50 μl nuclease-free water.

Detection of *P. fijiensis* and biomass estimation from samples (i.e., pure culture, non-inoculated, and inoculated) were determined by qPCR and calculating sample DNA (Threshold cycle) *C*t mean values, to generate an equation *Y* = -0.265*x* + 6.0582 from linear regression curve. *Y* is defined as concentration and *X* is the *C*t values. The reaction mixture and conditions for qPCR remained as described in gene expression assay above, except here we used β- tubulin primers for *P. fijiensis* detection and 100 ng/μl DNA in 12 μl total reaction volume.

In this experiment, three silenced strains of *PfFus3* and *PfSlt2* were selected as biological replicates including three technical replicates and experiments were repeated thrice.

### Statistical Data Analysis

The data were analyzed using GenStat 7th Edition statistical software package employing ANOVA to test significance differences, comparison of means and total mean necrosis in the silenced transformants and wild type.

## Results

### Confirmation of *Pseudocercospora fijiensis* Isolates

The pure cultures of *P. fijiensis* isolated from infected leaves of the banana cultivar ‘Nakitembe’ were confirmed by PCR analysis. An amplicon of the expected size of 1000 bp was obtained similar to control *P. fijiensis* collected from CBS-KNAW Fungal Biodiversity Centre in Netherlands, confirming their identity to be *P. fijiensis* (**Supplementary Figure S1**). However, *P. musae*, *P. eumusae* and non-template used as controls did not show any amplification.

### Plasmid Construct Preparation

Two RNAi plasmid constructs were prepared by cloning independently *PfFus3* or *PfSlt2* into dual promoter pKOIISD1, a silencing vector for fungal pathogen. The promoter Ptrpc drives sense and Pgpd drives antisense sequences of the target genes (i.e., *PfFus3* and *PfSlt2*) to generate dsRNA (**Figures 2A,B**). Both the plasmid constructs were confirmed by PCR for presence of insert and sequencing for orientation of insert. The orientation of both *PfSlt2* and *PfFus3* genes was confirmed to be as 5′ to 3′ (sense strand) for the Ptrpc promoter and 3′ to 5′ (antisense) for the Pgpd promoter.

### Generation and Molecular Characterisation of Transformed *P. fijiensis*

**FIGURE 3:**
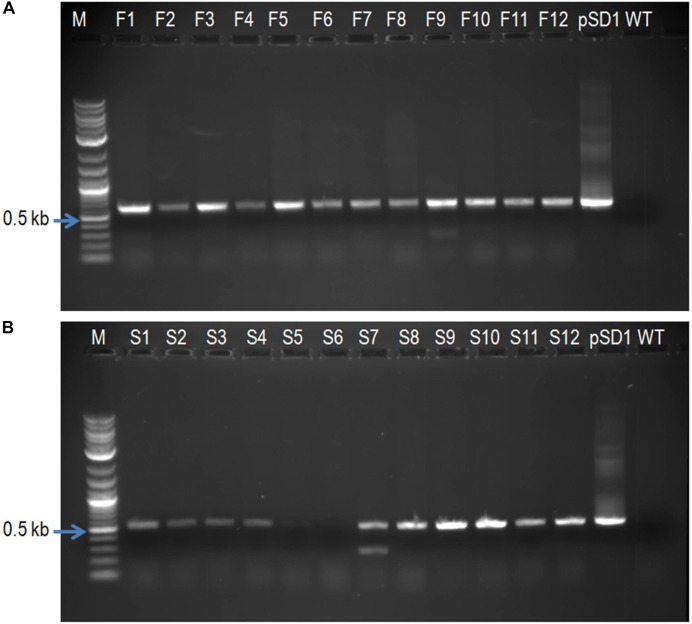
PCR analysis of silenced transformants of *P. fijiensis*. **(A)** *PfFus3* lane F1–F12 contains inserts; **(B)** *PfSlt2* lane S1–S4, S7–S12 all confirmed presence of inserts except lane S5 and S6. M, molecular weight marker; pSD1, pKOIISD1 vector containing *PfFus3* or *PfSlt2* used as a positive control; WT, wild type, non-transformed *P. fijiensis*.

The *P. fijiensis* transformants were generated through *Agrobacterium tumefaciens* mediated transformation and validated by PCR analysis. PCR analysis revealed presence of insert with an expected size (based on plasmid construct map) of 613 bp amplicon in all the *PfFus3* transformants tested (**Figure 3A**). As seen in **Figure 3B**, the *PfSlt2* transformants showed a product of expected size of 520 bp except for two transformants (lane S5 and S6). The non-transformed control *P. fijiensis* (WT) did not show any amplified products.

**FIGURE 4:**
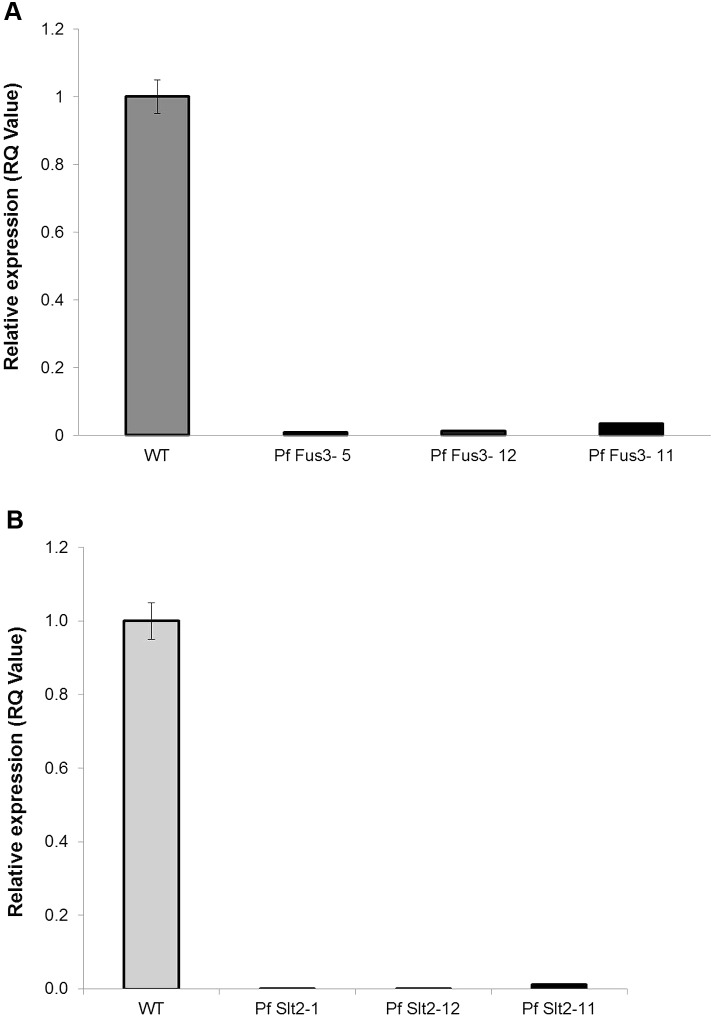
Relative expression (RQ) of *Fus3* and *Slt2* in silenced strains of *P. fijiensis* compared to wild type (WT). **(A)** *PfFus3* transformants (*Pf Fus3-5, Pf Fus3-12, Pf Fus3-11)*; **(B)** *PfSlt2* transformants (*PfSlt2-1, PfSlt2-12, PfSlt2-11).* Three technical replicates of each mutant and WT control were used in each experiment. The experiment was repeated thrice and data are presented as Mean ± SE.

Silencing of *PfFus3* and *PfSlt2* genes in *P. fijiensis* transformants was confirmed by qRT-PCR assays and relative (*C*_t_) Quantitation. The specificity of the primers used in the qRT-PCR assays was verified by generating a linear regression curve using absolute quantitation (**Supplementary Figure S2**). The *R* square (*R*^2^) and efficiency of the primer pair used for the amplification of β*-tubulin* was 0.9981 and 104.8%, respectively, where as *R*^2^ and efficiency of primer pairs used for amplification of the *PfFus3* was 0.9960 and 104% and *PfSlt2* was 0.9961 and 104%, respectively. Relative expressions of *Fus3* in *PfFus3* mutant strains were 0.008 (0.8%), 0.0133 (1.33%), 0.0346 (3.46%), respectively, for *PfFus3- 5*, *PfFus3-12*, and *PfTFus3-11* in comparison to the wild-type control (100%) (**Figure 4A**). Similarly, the relative expression levels of *Slt2* in *PfSlt2* transformants were 0.00085 (0.085%), 0.001188 (0.119%), 0.0128 (1.28%) for *PfSlt2-1*, *PfSlt2-12*, and *PfSlt2-11*, respectively, in relation to the wild type (100%) (**Figure 4B**).

These results confirmed that silencing of *PfFus3* and *PfSlt2* in *P. fijiensis* reduced expression by more than 95% (*Fus3* range from 96.54 to 99.2%, *Slt2* from 98.77 to 99.915%) in comparison to wild-type control. The expression of *PfFus3* and *PfSlt2* genes was nearly undetectable in most of the transformed *P. fijiensis*.

### Virulence Assays for *PfFus3* and *PfSlt2 P. fijiensis* Transformants on Host Plants

**FIGURE 5:**
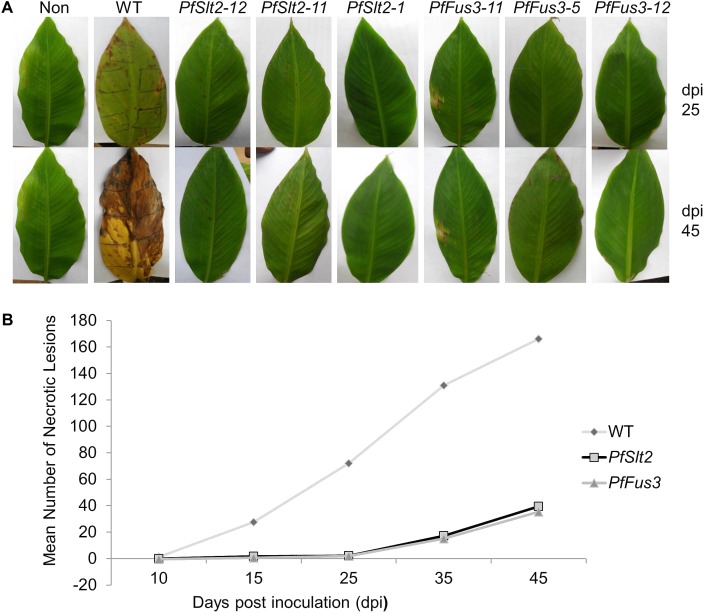
Effect of silencing of *PfFus3* and *PfSlt2* genes on the disease development in banana cultivar ‘Nakitembe’ inoculated with *P. fijiensis*. **(A)** Plant leaves (abaxial side) showing disease symptom progression (i.e., necrosis) at 25 and 45 days post inoculation (dpi). Leaves inoculated with 10% rich medium containing 1% tween 20 acted as non-inoculated control (NIC). Leaves were inoculated with wild-type strain of *P. fijiensis* (WT) and three independent *PfFus3* transformants (*PfFus3-5, PfFus3-11, PfFus3-12*) and *PfSlt2* transformants (*PfSlt2-1*, *PfSlt2-11, PfSlt2-12*). **(B)** Summary representation of total mean necrosis in plant leaves inoculated with WT, transformants *PfFus3* and *PfSlt2* across different days post inoculation.

The effect of silencing of *PfFus3* and *PfSlt2* on pathogenicity of *P. fijiensis* was determined by inoculating leaves of banana plants of the EAHB cultivar ‘Nakitembe’ with mycelia of *PfFus3* and *PfSlt2* transformants and the wild-type control. Plants inoculated with the wild-type control strain developed disease symptoms between 9 and 10 days post inoculation (dpi). However, development of disease symptoms in plants inoculated with *PfSlt2* transformants was delayed and was apparent at 15 dpi, while plants infected with *PfFus3* transformants showed disease symptoms between 18 and 19 dpi (**Figure 5**).

Symptom development and disease progression was faster in plants inoculated with wild-type *P. fijiensis* compared to the *PfFus3* and *PfSlt2* transformants. This is shown by the higher levels of necrosis in plants infected with wild type at 25 and 45 dpi (**Figures 5A,B**). There was a significant difference (*P* < 0.001) in total mean necrosis between the plants infected with wild type and transformants. The percentage mean necrosis at 45 dpi in plant leaves inoculated with wild type strain was 79.6% (166.1), which was three times higher than the necrosis in plant leaves inoculated with transformed strains of *PfFus3* 10.5% (35.3) and *PfSlt2* 12.1% (39.4) (**Figure 5B**). The *PfFus3* transformants were significantly (*p* < 0.001) less virulent than the *PfSlt2* transformants.

### Role of *PfFus3* and *PfSlt2* in Invasive Growth of *P. fijiensis*

**FIGURE 6:**
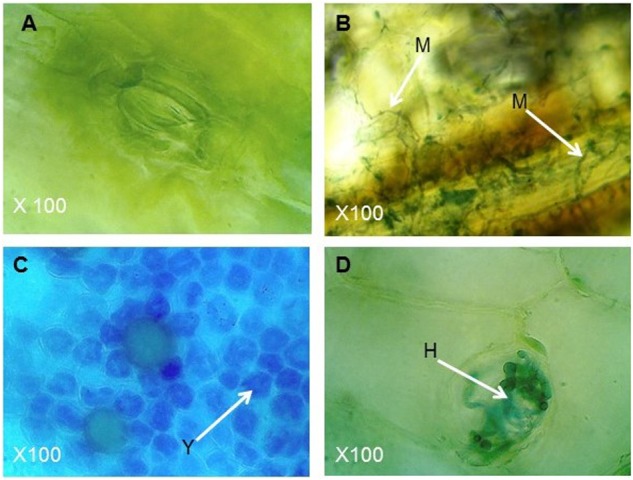
*Pseudocercospora fijiensis* mycelia growth and colonization in leaf tissue stained with lacto phenol cotton blue. **(A)** Non-inoculated leaf tissue with no evidence of fungal growth; **(B)** Leaf tissue inoculated with wild-type *P. fijiensis* showing intercellular mycelial growth (arrow M); **(C)** Leaf tissue inoculated with *PfFus3* transformants showing clamps of hyphae (arrow Y); **(D)** Leaf tissue inoculated with *PfSlt2* transformants showing restricted swollen dormant state hyphae in inter cellular space (arrow H).

To confirm the role of *PfFus3* and *PfSlt2* for invasive growth of *P. fijiensis*, leaf tissues inoculated with the *PfSlt2* and *PfFus3* transformants along with non-inoculated tissues as negative control and leaf tissues inoculated with wild-type *P. fijiensis* as a positive control were stained with Lacto phenol cotton blue. No mycelium was observed in non-inoculated tissues (**Figure 6A**). However, the wild-type *P. fijiensis* colonized the leaf tissue as shown by staining with the lacto phenol cotton blue (**Figure 6B**). Leaf tissues inoculated with *PfFus3* transformants revealed aggregation of mycelia in necrotic leaf tissues without any growth in intercellular spaces (**Figure 6C**). Similarly, *PfSlt2* transformant-inoculated tissues showed deformed swollen knob mycelia structure with no invasive growth in the intercellular spaces (**Figure 6D**).

### Measurement of Mycelia Growth of *P. fijiensis* in Leaf Tissues

The presence of *P. fijiensis* in the leaf tissue inoculated with wild-type and transformants was confirmed by PCR amplification using primers specific to β*-tubulin* gene and based on the ITS region of the fungus. The expected size of the amplicons were observed to be similar to the pure culture of *P. fijiensis* collected from CBS-KNAW Fungal Biodiversity Centre in Netherlands, confirming their identity to be *P. fijiensis* (**Supplementary Figures S3A–C**). No amplification was noticed in the non-inoculated leaf tissue.

**FIGURE 7:**
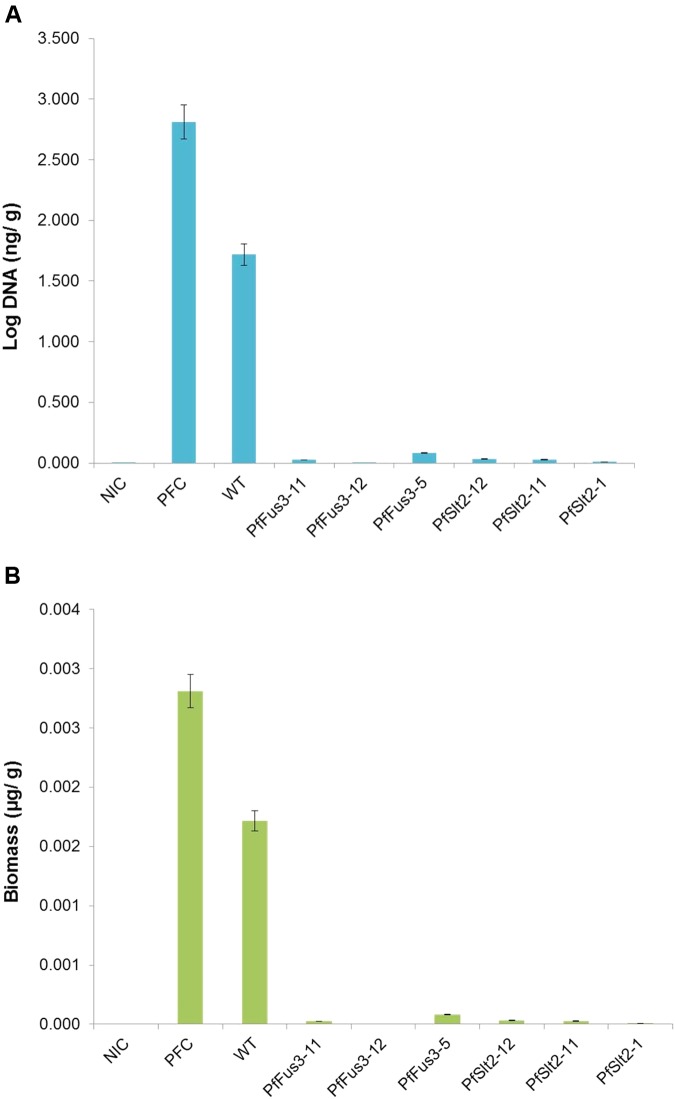
Estimates of DNA and biomass of *P. fijiensis* in different banana leaf samples inoculated with wild type and transformants. **(A)** Fungal DNA estimates; **(B)** Biomass estimates. NIC, DNA from non-inoculated leaf; PFC, DNA from pure culture of *P. fijiensis*; WT, DNA of leaf inoculated with wild-type *P. fijiensis*. Test sample DNA from leaves inoculated with *PfSlt2* transformants (*PfSlt2-1, PfSlt2-11, PfSlt2-12*) and *PfFus3* transformants (*PfFus3-5, PfTFus3-11, PfFus3-12*).

Colonization or invasive growth of *P. fijiensis* in tissue to evaluate pathogenicity was further confirmed through biomass quantification. The leaves inoculated with wild-type *P. fijiensis* showed a high amount of fungal DNA (1.718 ng/g), confirming the presence of high fungal biomass. However, leaf tissues inoculated with silenced *PfFus3* and *PfSlt2* transformants showed extremely low *P. fijiensis* DNA concentrations; this varied between 0.000727 to 0.0250 ng/g and 0.0098 to 0.0322 ng/g, respectively (**Figures 7A,B** and **Table 1**). As expected, no fungal DNA was detected in non-inoculated leaf tissue controls while positive control pure cultures of *P. fijiensis* gave high biomass.

**Table 1:** Measurement of fungal growth in different banana samples inoculated with wild-type and transformants of *P. fijiensis.*

Sample details	Sample ID	*C*t mean	*C*t mean (×10^1^)	DNA Concentration (ng/g)	Biomass (μg/g)
DNA of non-inoculated banana leaf	NIC	33.30	3.33	0.000762	7.62 × 10^-7^
DNA of pure culture of *P. fijiensis*	PFC	20.66	2.07	2.8119	2.81 × 10^-3^
DNA of banana leaf inoculated with wild-type *P. fijiensis*	WT	21.42	2.14	1.718	1.72 × 10^-3^
DNA of banana leaf inoculated with *PfFus3* transformants	*PfFus3-11*	27.91	2.79	0.0250	2.5 × 10^-5^
	*PfFus3-12*	33.37	3.34	0.000727	7.27 × 10^-7^
	*PfFus3-5*	26.11	2.61	0.0816	8.16 × 10^-5^
DNA of banana leaf inoculated with *PfSlt2* transformants	*PfSlt2-12*	27.54	2.75	0.0322	3.22 × 10^-5^
	*PfSlt2-11*	27.78	2.78	0.0276	2.76 × 10^-5^
	*PfSlt2-1*	29.36	2.94	0.0098	9.8 × 10^-6^


The relationship between the amount of biomass estimate per sample and threshold cycle (*C*t) values were also estimated. High *C*t values were observed in non-inoculated and silenced *PfSlt2*, *PfFus3* infected samples. However, *P. fijiensis* pure culture and samples infected with wild-type *P. fijiensis* showed very low *C*t values. This confirms that high *C*t values relate to low fungal biomass and low *C*t values is an indication of high *P. fijiensis* biomass (**Table 1**).

## Discussion

MAP kinase Fus3 and Slt2 pathways are known to be responsible for regulating host penetration, infectious, invasive growth, and pathogenicity of several fungal pathogens including *Mycosphaerella graminicola* (Mehrabi et al., 2006; Cousin et al., 2007). A key interest of this study was to assess the importance of *Fus3* and *Slt2* homolog genes of *P. fijiensis* in infection processes and pathogenicity.

The fragments of MAP kinase genes *PfSlt2* and *PfFus3* were cloned into the RNAi vector pKOIISD1. The transformed *P. fijiensis* carrying *Slt2* and *Fus3* was confirmed by end point PCR assays. We further showed decreased gene expression effects by Relative Quantitation (RQ) using qRT-PCR. The expression of *Fus3* and *Slt2* genes in *P. fijiensis* transformants was significantly reduced compared to expression in the wild-type strain. RNA-interference-mediated gene silencing proved to be highly efficient as demonstrated by the nearly undetectable expression of *Fus3* and *Slt2* in the *PfFus3* and *PfSlt2* silenced strains. This concurred with previous studies which showed that silencing of endogenous gene *Mpg1* and polyketide synthase-like gene in *Magnaporthe oryzae* using pSilent-1 led to reduction in expression level by 70–90% (Nakayashiki et al., 2005). This study demonstrated that RNAi-mediated gene silencing being a good tool for the study of gene functions in fungal pathogens including *P. fijiensis*.

The molecular mechanism behind virulence has been studied in *Candida glabrata* (Miyazaki et al., 2010) and a few fungal plant pathogens such as *U. maydis* (Mayorga and Gold, 1999), *F. proliferatum* (Zhao et al., 2012), and *Colletotrichum higginsianum* (Wei et al., 2016). However, in the ascomycete *P. fijiensis* the perception of host signaling, penetration, and colonization of the host plant tissues is unknown. Most especially the role of MAP kinase encoding genes *Fus3* and S*lt2* as pathogenicity factors in *P. fijiensis* are not known.

Several previous studies identified *Fus3* and S*lt2* as important genes in regulating pathogenesis factors and pathogenicity in other fungal pathogens (Miyazaki et al., 2010; Luo et al., 2012; Li et al., 2014). Here, we investigated the role of *Fus3* and *Slt2* in the pathogenicity of *P. fijiensis* on banana plants, and to the best of our knowledge, this is the first study reporting the importance of *Fus3* and *Slt2* in invasive growth and pathogenicity of *P. fijiensis*. The silenced strains of *PfFus3* and *PfSlt2* showed less virulence characterized by reduced efficiency of plant infection, reduced invasive growth, and fewer necrotic symptoms on “susceptible” EAHB cultivar ‘Nakitembe’. Symptom development in plants inoculated with silenced strains of *PfFus3* and *PfSlt2* was delayed by only few days as compared to plants inoculated with wild-type strain. However, progression of symptoms and colonization of fungi were significantly impaired. This implies that these genes play minor roles in initial symptom development but are critical in regulating fungal development processes like invasive growth of *P. fijiensis* and plant infection. Previous studies also reported that MAPKs pathways regulate growth and development of other fungal pathogens (Alonso-Monge et al., 2001). Interestingly, there was no evidence of invasive growth in leaf tissues inoculated with *PfSlt2* and *PfFus3* silenced transformants when tissues were examined microscopically.

Our findings are supported by earlier research which showed that MAP kinase *Slt2* and their homologs contribute to invasive growth of *Mycosphaerella graminicola* (Mehrabi et al., 2006), colonization in host tissue (Rui and Hahn, 2007), and virulence in *Phytophthora sojae, Beauveria bassiana, Candida glabrata, Colletotrichum higginsianum* (Miyazaki et al., 2010; Luo et al., 2012; Li et al., 2014; Wei et al., 2016). Furthermore, *PfSlt2* transformants failed to invasively grow and colonize cells but remained as swollen dormant thalli. It is possible that *PfSlt2* transformants failed to adapt to the intercellular environment leading to the failure to develop sufficient turgor pressure to penetrate cells from intercellular space. MAP kinase *Slt2* is well known to be responsible for the fungal cell wall integrity (Mehrabi et al., 2006), thus *PfSlt2* transformants could have lost the ability to maintain cell wall integrity in the intercellular space. Similarly, *Slt2* homolog in *Alternaria alternata* (*AaSlt2*) was shown to be critical for cell-wall integrity, responsible for hyphal elongation. *AaSlt2* transformants produced globose, swollen hyphae, and failed to elongate (Yago et al., 2011). The formation of swollen hyphal structures in leaves inoculated with *PfSlt2* transformants is an indication of retarded growth and hyphal deformation. It is an indication that *PfSlt2* regulates key factors critical for pathogenicity of *P. fijiensis*.

The *PfFus3* transformants formed undifferentiated massive aggregation of mycelia in necrotic tissues suggesting that fungal development was arrested at an early stage, thereby impairing intercellular hyphal growth. Homologs of *Fus3* are known to be essential for infection processes like formation of infection structures, sporulation, invasive and filamentous growth and virulence in other fungal pathogens such as *Metarhizium acridum, Colletotrichum higginsianum*, and *F. proliferatum* (Zhao et al., 2012; Jin et al., 2014; Wei et al., 2016). Similar observations were demonstrated by silenced *PfFus3*, confirming the role of *Fus3* in infection processes and pathogenicity of *P. fijiensis.*

Lastly, low fungal-biomass estimates in leaf tissue infected with *PfFus3* and *PfSlt2* mutant strains demonstrated that colonization and invasive growth was at least partly regulated by *PfFus3* and *PfSlt2* in *P. fijiensis*. There was also a positive correlation between *C*t value and biomass estimate, meaning high *C*t values indicate low biomass while a low *C*t value is an indication of high biomass. This greatly complements screen-house visual pathogenicity assay assessments and Lacto phenol cotton blue staining assays for estimating fungal biomass. A similar study was used to quantify growth of *F. graminearum* and *Magnaporthe oryzae in planta* fungal pathogenicity (Qi and Yang, 2002; Horevaj et al., 2011). This study clearly demonstrates that MAP kinase *Fus3* and *Slt2* pathways are significant contributors to plant infection and growth of *P. fijiensis* in infected plant tissues. Similarly, several previous studies showed that the MAP kinases *Fus3* and *Slt2* are involved in the formation of infection structure, spore formation, pathogenic growth or colonization, and pathogenicity. For example *Fus3/Kss1* homolog *FPK1* in *F. proliferatum* is involved in hyphal growth, conidiation, and plant infection (Zhao et al., 2012). In *Phytophthora sojae PsMPK1* a homolog of *Slt2* is known to be required for hyphal growth, zoosporogenesis, cell-wall integrity, and pathogenicity (Li et al., 2014).

Despite efforts to investigate the role of *PfSlt2* and *PfFus3* in the pathogenicity of *P. fijiensis* in this study, the molecular interaction between banana and *P. fijiensis* is not yet well understood. However, previous studies showed that some *Musa* accessions are highly resistant to the BSD, as the mortality of the host cells occurs fast after infection avoiding and preventing the spread of the pathogen into the rest of the plant (Lepoivre et al., 2003). The resistance in *Musa* against *P. fijiensis* is obtained after stomata penetration due to hypersensitive reaction or antifungal activity of phytoalexins and structural analogs (Hoss et al., 2000; Quiñones et al., 2000; Lepoivre et al., 2003). The wild diploid accession ‘Calcutta 4’ is known to be resistant to BSD because of the expression of pathogenesis related proteins especially during the infection process by *P. fijiensis* (Rodriguez et al., 2016). ‘Calcutta 4’ also seems to have some unknown resistance genes that recognizes the PfAVR4 protein, which resulted in hypersensitive reaction upon infiltration of PfAVR4 protein into the banana leaves (Arango Isaza et al., 2016). The disease resistance genes from resistant *Musa* accessions could be transferred to susceptible bananas and plantain cultivars through conventional breeding or genetic engineering. However, a deeper understanding of the genes involved in fungal resistance process is required.

In summary, the role of MAP kinase *Fus3* and *Slt2* genes in the pathogenicity and growth in the host plant has been demonstrated in several fungal pathogens, now including *P. fijiensis.* It has been reported that fungal genes responsible for pathogenicity could be silenced through host-induced gene silencing (HIGs) to develop disease-resistant plants. Example transgenic wheat with resistance against *Blumeria graminis* and transgenic banana with resistance to fusarium wilt disease (Nowara et al., 2010; Ghag et al., 2014). Therefore, findings from this study suggest that BSD might be controlled by developing transgenic banana-targeting silencing of *PfFus3* and *PfSlt2* in *P. fijiensis* through host induced gene silencing.

## Author Contributions

FO developed the research concept, conducted the experiments, collected and analyzed the data, and wrote the manuscript. GT shaped the research concept and guided and supervised the experiments. L-HC supported vector design, gene cloning, and transformation of fungi. BF and IS shaped the research concept and guided and supervised experiments. JT supported gene expression assay and microscopy. WT and JK provided research supervision and LT shaped the research concept, guided and supervised the experiments, and wrote the manuscript. All authors reviewed and edited the manuscript.

## Conflict of Interest Statement

The authors declare that the research was conducted in the absence of any commercial or financial relationships that could be construed as a potential conflict of interest.

## Supplementary Material

**FIGURE S1 |** PCR analysis of *P. fijiensis* isolated from infected leaves of banana plant using *P. fijiensis* specific primers based on ITS region (Johanson and Jeger, 1993). Lanes 1–3- *P. fijiensis* isolates, Pf- *P. fijiensis* collected from Fungal Biodiversity Centre, Netherlands as positive control, Pm- *P. musae* collected from Fungal Biodiversity Centre, Netherlands, Pe- *P. eumusae* collected from Fungal Biodiversity Centre, Netherlands, NT- non-template control.

**FIGURE S2 |** Linear regression curves confirming qPCR primer efficiency. **(A)** β*-tubulin*; **(B)** *PfFus3*; **(C)** *PfSlt2*.

**FIGURE S3 |** PCR amplification of DNA isolated from leaf tissues inoculated with *P. fijiensis* transformants. **(A)** PCR amplification of *PfSlt2* transformants (S1, S2, S3) using primers specific to β*-tubulin* gene; **(B)** PCR amplification of *PfFus3* transformants (F1, F2, F3) using primers specific to β*-tubulin* gene; **(C)** PCR amplification of *PfFus3* transformants (F1, F2, F3) and *PfSlt2* transformants (S1, S2, S3) using *P. fijiensis* specific primers based on ITS region (Johanson and Jeger, 1993). P- pure culture of *P. fijiensis* collected from Fungal Biodiversity Centre, Netherlands as positive control, WT- tissues inoculated with wild-type *P. fijiensis*, NC- non-inoculated control tissue.
