# Supplementary figures and images for: Silencing of the Mitogen-Activated Protein Kinases (MAPK) Fus3 and Slt2 in Pseudocercospora fijiensis Reduces Growth and Virulence on Host Plants
Source: Front Plant Sci. 2018 Mar 13;9:291. doi: 10.3389/fpls.2018.00291 (PMC5859377; doi:10.3389/fpls.2018.00291)

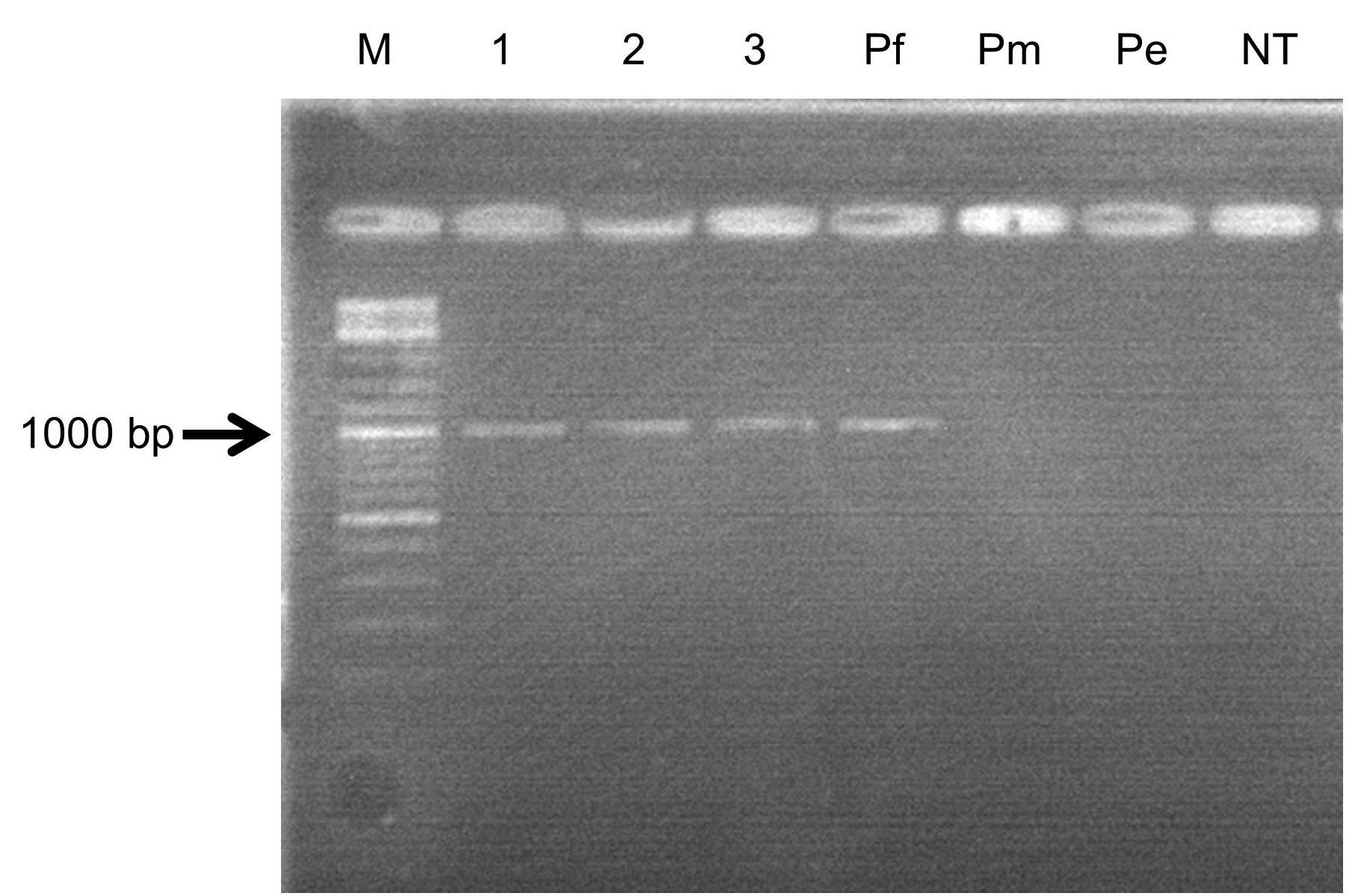

Supplement: Supplementary file 1 [file Image_1.JPEG]

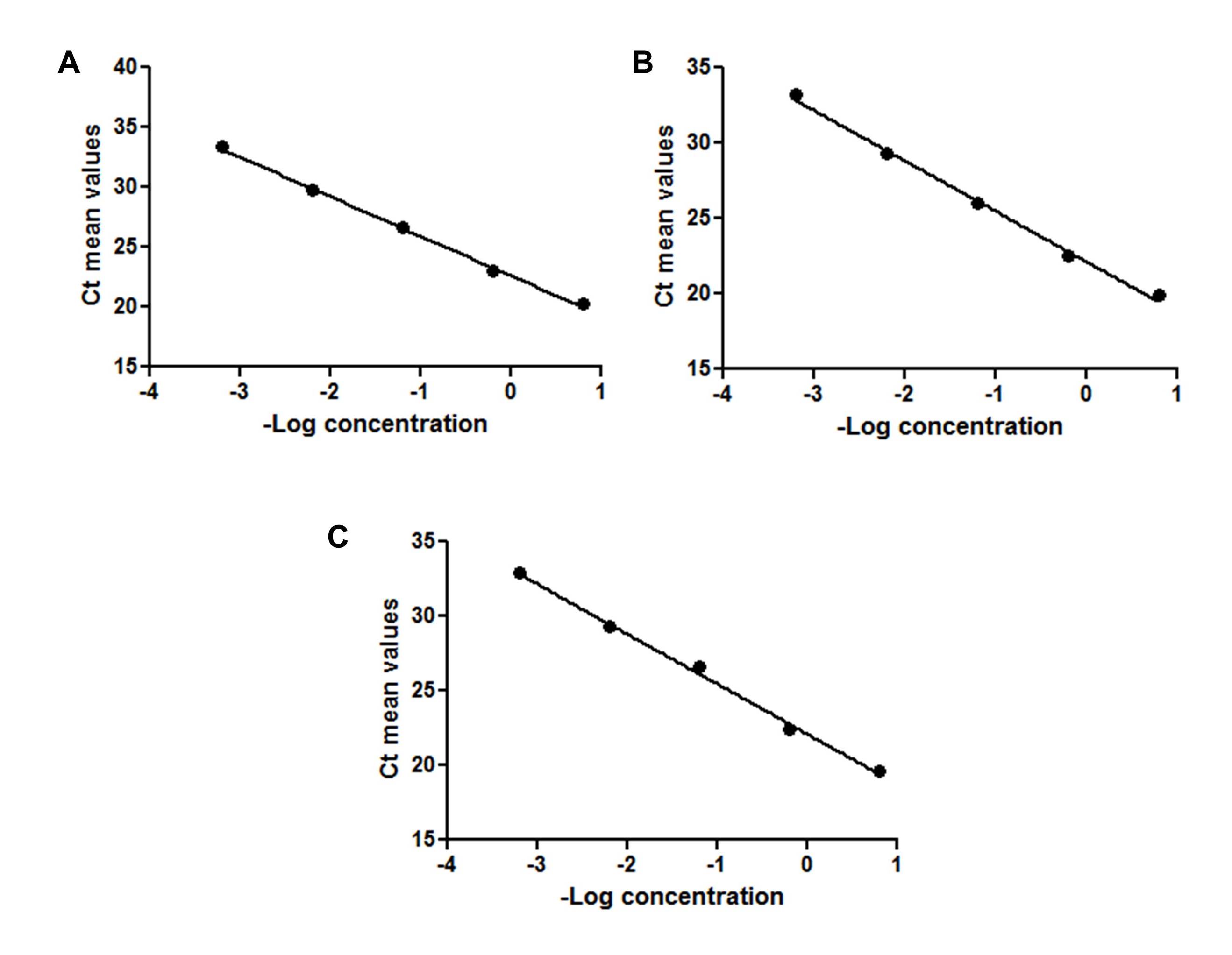

Supplement: Supplementary file 2 [file Image_2.JPEG]

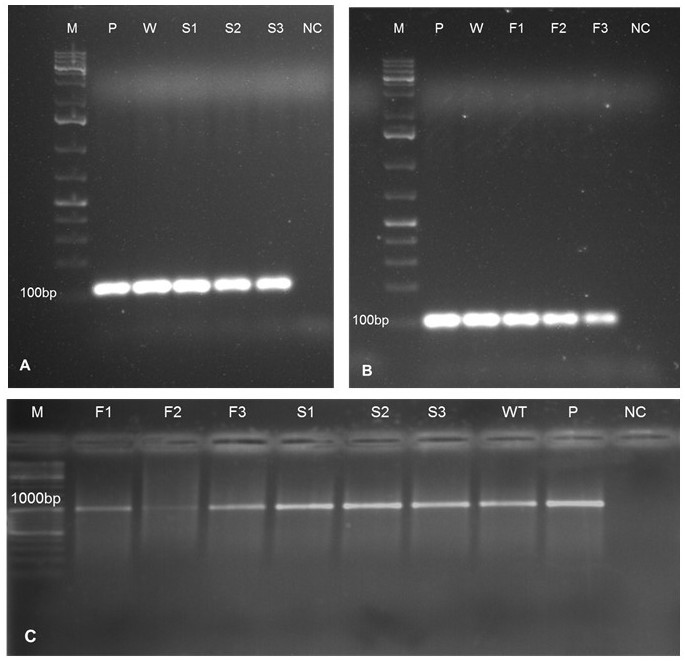

Supplement: Supplementary file 3 [file Image_3.JPEG]
